# Ambient cold exposure and myocardial infarction risk in Tianjin, China: a distributed lag non-linear analysis

**DOI:** 10.1186/s12889-026-28924-7

**Published:** 2026-08-07

**Authors:** Tingting Li, Lin Wang, Yixuan Yin, Hongliang Cong

**Affiliations:** 1 Department of Cardiology, Tianjin Chest Hospital, Tianjin, China; 2 Department of Geriatrics, The Second Hospital of Tianjin Medical University, Tianjin, China; 3 Internal Medicine of Traditional Chinese Medicine, Tianjin University of Traditional Chinese Medicine, Tianjin, China

**Keywords:** Acute myocardial infarction, Distributed lag non-linear model, Extreme cold, ST-segment elevation myocardial infarction, Non-ST-segment elevation myocardial infarction

## Abstract

**Background:**

Ambient temperature is an important environmental determinant of cardiovascular health. Although cold exposure has been associated with acute myocardial infarction (MI), evidence on delayed effects and subtype-specific differences remains limited in northern China. We examined the short-term association between temperature and MI incidence in Tianjin.

**Methods:**

We conducted a time-series study integrating daily MI counts from the Tianjin Chest Pain Center with meteorological data from 2020 to 2023. Distributed lag non-linear models with quasi-Poisson regression were used to estimate non-linear and lagged effects of temperature on MI, adjusting for long-term trends, seasonality, relative humidity, day of the week, and public holidays. Extreme cold was defined as the 5th percentile of daily mean temperature (− 3.4 °C). The minimum morbidity temperature (MMT) was used as the reference temperature.

**Results:**

During the 1,461-day study period, 34,940 MI events were recorded, including 21,814 (62.4%) STEMI cases. Compared with the median temperature (15.4 °C), extreme cold was associated with a cumulative 7-day RR of 1.53 (95% CI: 1.25–1.87), with peak effects observed at lag 2 days. The cumulative risk appeared higher for STEMI (RR = 1.62, 95% CI: 1.31–2.01) than NSTEMI (RR = 1.38, 95% CI: 1.05–1.82), but this difference was not statistically significant (*P*_heterogeneity_ = 0.367). Referenced to the MMT, the temperature–MI association showed a non-linear, approximately U-shaped pattern, with extreme cold associated with increased risk (RR = 1.66, 95% CI: 1.35–2.04). The bootstrap-derived MMT was 24.1 °C (95% CI: 22.6–26.2 °C), consistent with the primary estimate.

**Conclusions:**

Ambient cold exposure is associated with a short-term increase in MI risk in Tianjin, peaking approximately 2 days after exposure. Although STEMI showed higher descriptive risk than NSTEMI, no statistically significant difference was observed. Heat-related associations were not statistically significant and should be interpreted cautiously due to limited exposure frequency and statistical power. These findings provide evidence for cold-related cardiovascular risk in temperate regions.

**Clinical trial registration:**

Not applicable.

**Supplementary Information:**

The online version contains supplementary material available at https://doi.org/10.1186/s12889-026-28924-7.

## Introduction

Cardiovascular disease (CVD) remains the leading cause of death worldwide and continues to impose a major public health burden [1]. Ambient temperature is an important environmental determinant of cardiovascular health, and increasing evidence suggests that temperature-related exposures may trigger acute cardiovascular events [2]. Among these, myocardial infarction (MI) is of particular concern because of its high incidence, severity, and contribution to premature mortality.

Epidemiological studies have linked both cold and heat exposure to MI risk, but the magnitude, timing, and shape of these associations vary across settings [3, 4]. In Sweden, short-term changes in ambient temperature were associated with MI hospital admissions, with delayed increases observed after temperature decreases [3]. A recent systematic review and meta-analysis also found that cold exposure was associated with adverse cardiovascular outcomes, while highlighting substantial heterogeneity across climatic regions and study designs [4]. This variability limits the direct generalizability of findings and highlights the need for locally specific evidence.

In addition to cold exposure, high ambient temperatures have also been associated with increased risk of myocardial infarction and other cardiovascular outcomes, although evidence remains more heterogeneous across populations and climatic regions. Large-scale multi-country studies have suggested that both extreme heat and extreme cold contribute to cardiovascular morbidity and mortality, supporting a potential non-linear (U-shaped or J-shaped) temperature–health relationship [5].

Cold exposure may increase cardiovascular risk through multiple pathways, including vasoconstriction, sympathetic activation, elevated blood pressure, and increased cardiac workload [6]. These effects are particularly relevant because cold exposure is common, seasonal, and potentially preventable through early warning systems and targeted protection of vulnerable populations. Previous studies have also suggested that cold-related cardiovascular effects may persist for several days and may be more pronounced in susceptible groups such as older adults [3, 7]. For example, a study in Dezful, Iran, reported increased cardiovascular hospitalizations during cold waves, particularly among older individuals [7].

Evidence from northern China remains limited despite the strong relevance of cold exposure to population health in this region. Tianjin serves as a representative setting for examining these associations. As a coastal megacity with over 13 million residents, Tianjin experiences a temperate monsoon climate characterized by cold winters and pronounced day-to-day temperature variability. The burden of ischemic heart disease and acute coronary events in northern China is also among the highest nationwide, highlighting the importance of region-specific evidence. Evaluating the temperature–cardiovascular relationship in this setting is methodologically advantageous due to high population density and relatively stable environmental monitoring conditions, which reduce exposure measurement variability. Evidence from Europe and the Middle East provides important context [3, 7], but may not be fully generalizable to Chinese cities due to differences in climate, infrastructure, and population characteristics.

In this study, we analyzed daily MI counts from the Tianjin Chest Pain Center (CPC) together with meteorological data from January 2020 to December 2023. Using distributed lag non-linear models(DLNM) with quasi-Poisson regression, we examined the non-linear and delayed associations between ambient temperature and MI incidence after adjustment for long-term trend, seasonality, relative humidity, day of the week, and public holidays. We also assessed subtype-specific patterns for ST-segment elevation myocardial infarction (STEMI) and non-ST-segment elevation myocardial infarction (NSTEMI), anchoring extreme cold at the 5th percentile (− 3.4 °C) and identifying the minimum morbidity temperature (24.5 °C) as the local optimum reference. Our aim was to provide evidence relevant to cold-related cardiovascular prevention and public health planning in northern China.

## Methods

### Study design and setting

This ecological time-series study evaluated the short-term association between ambient temperature and daily MI incidence in Tianjin, China, from 1 January 2020 to 31 December 2023. Population-level associations were assessed by linking daily MI counts with corresponding meteorological data on the same calendar days.

MI cases were identified from the Tianjin CPC registry. Records with a primary diagnosis of acute myocardial infarction (ICD-10 I21) were included. Cases were limited to residents of Tianjin or individuals with verified local onset to reduce potential exposure misclassification. Records with missing key diagnostic or temporal information were excluded. The final dataset was aggregated into daily counts and stratified into STEMI and NSTEMI for analysis.

The Tianjin CPC Registry is part of a city-wide, Ministry-accredited CPC network administered by the Tianjin Municipal Health Commission. Tianjin has a permanent resident population of approximately 13.6 million across 16 urban, peri-urban, and rural districts. During the study period, 32 CPC-certified secondary and tertiary hospitals were integrated into a unified acute coronary syndrome (ACS) care pathway.

These hospitals are connected to approximately 270 township/community Chest Pain Treatment Units and 468 village/clinic Chest Pain Points through a three-tier referral system with standardized emergency medical service prenotification and pre-hospital electrocardiogram transmission, enabling systematic routing of suspected ACS cases into the CPC network.

### Ethical approval

The study protocol was approved by the Ethics Committee of Tianjin Chest Hospital (Approval No. 2026LW-027) and was conducted in accordance with the Declaration of Helsinki. Because this was a retrospective study based on routinely collected de-identified data, the requirement for written informed consent was waived by the Ethics Committee.

### Health outcome data and pre-processing

Daily MI data were obtained from the Tianjin CPC electronic registry. Diagnoses were based on standardized clinical criteria using ICD-10 coding (I21) and registry protocols. The data were aggregated into a 1,461-day time-series dataset, with daily counts generated for total MI and further stratified into STEMI and NSTEMI according to guideline-based definitions [8].

The registry captures hospital-managed MI cases within the CPC network but does not include out-of-hospital cardiac deaths, pre-hospital fatal myocardial infarction, or cases treated outside CPC-certified hospitals; therefore, it reflects hospital-presenting MI events rather than complete population-level incidence.

All days recorded at least one MI event (zero-count days: 0%), and therefore quasi-Poisson regression was applied without zero-inflation modeling.

### Environmental exposure assessment

Continuous daily meteorological observations for Tianjin were obtained from the National Centers for Environmental Information (NCEI) of the National Oceanic and Atmospheric Administration (NOAA). Data were restricted to the Tianjin National Reference Station (WMO ID: 54527) covering 1 January 2020 to 31 December 2023 (1,461 days). The dataset included daily mean, maximum, and minimum temperatures, relative humidity, and wind speed. The primary exposure variable was daily mean temperature (T_mean_).

Two temperature references were defined: (1) median temperature (15.4 °C) as the baseline reference; and (2) minimum morbidity temperature (MMT), defined as the temperature associated with the lowest cumulative relative risk over lag 0–7 days estimated from the DLNM. Extreme cold was defined as the 5th percentile (− 3.4 °C).

The MMT was estimated by predicting cumulative relative risks across the observed temperature range and identifying the minimum point. Uncertainty was quantified using a non-parametric bootstrap procedure (500 resamples), in which the DLNM was refitted in each sample. The 95% CI was derived from the empirical distribution of bootstrap estimates. The bootstrap-derived MMT was 24.1 °C (95% CI: 22.6–26.2 °C), which was consistent with the primary estimate (24.5 °C), indicating consistency between estimates. This positioning is consistent with previous multi-city epidemiological studies in China, which have shown that optimal thermal thresholds typically occur near the upper percentiles of local temperature distributions [9, 10]. A distribution plot of daily mean temperatures with the MMT indicated is provided in Supplementary Figure S1.

Missing meteorological data were rare (T_mean_: 0.07%; RH: 0.41%; T_max_: 1.44%; T_min_: 0.07%; wind: 1.23%). Missing values in T_mean_ (*n* = 1) and relative humidity (*n* = 6) were imputed using linear interpolation. T_max_, T_min_, and wind speed were used only for descriptive analyses.

Daily gaseous and particulate air pollution data (e.g., PM₂.₅, NO₂, O₃) were not available for the full study period and were therefore not included in the covariate adjustment. This may introduce residual confounding, given the known associations of air pollutants with both ambient temperature and acute cardiovascular events.

However, the direction and magnitude of this potential confounding are uncertain, as the correlation between temperature and air pollution varies by season, pollutant type, and local emission–meteorological conditions. Therefore, the impact of unmeasured air pollution on the estimated temperature–MI associations cannot be precisely quantified and should be interpreted as context-dependent.

Exposure was based on a single meteorological station, which may not fully capture spatial variability in temperature across Tianjin, but provides a stable proxy for city-wide exposure in this densely populated urban setting.

### Descriptive analysis

Descriptive statistics were used to summarize daily MI counts and meteorological variables during the study period. Continuous variables were presented as mean ± standard deviation for approximately normally distributed data and as median with interquartile range for skewed data. Spearman rank correlation coefficients were calculated to examine associations between daily MI counts and meteorological variables and to assess collinearity among environmental variables before model fitting.

### Statistical analysis

We used DLNMs combined with generalized linear models to assess the association between daily mean temperature and daily MI counts. Overdispersion was assessed using the Pearson chi-square statistic divided by the residual degrees of freedom, and quasi-Poisson regression was applied when dispersion exceeded 1.

A cross-basis function was specified to model the non-linear and lagged effects of temperature. Natural cubic splines with 3 degrees of freedom for temperature and 4 degrees of freedom for lag were used, guided by prior literature and model fit assessed using quasi-AIC (QAIC). A maximum lag of 7 days was selected a priori based on the biological plausibility of cold-triggered cardiovascular events. A sensitivity analysis extending the lag to 14 days showed consistent results.

The main model adjusted for potential temporal and meteorological confounding. Day of the week and public holidays were included as separate categorical variables in the regression model. Long-term trend and seasonality were controlled using a natural cubic spline of calendar time with 7 degrees of freedom per year, a specification chosen based on commonly used approaches in time-series epidemiological studies, which balance flexibility in capturing long-term and seasonal patterns while minimizing overfitting. Relative humidity was adjusted for using a natural cubic spline with 3 degrees of freedom.

Relative risks (RRs) and 95% confidence intervals (CIs) were estimated using the median temperature as the primary reference and the MMT as a secondary reference.

Subgroup analyses were conducted for STEMI and NSTEMI, estimating both lag-specific and cumulative effects.

Differences between STEMI and NSTEMI were assessed using a Wald-type heterogeneity test based on the method of Altman and Bland. Standard errors were derived from the 95% CIs as SE = [ln(upper CI) − ln(lower CI)] / 3.92. The test statistic was then calculated as: Z = [ln(RR_STEMI) − ln(RR_NSTEMI)] / √(SE_STEMI² + SE_NSTEMI²). A two-sided P value was obtained from the standard normal distribution [11].

### Sensitivity analyses

To assess the robustness of the main findings, we evaluated the stability of the estimated temperature–MI associations using alternative reference temperatures, including the median temperature and the MMT. Consistency in the direction and magnitude of the estimated cold effect across specifications was used to assess robustness. In addition, we extended the maximum lag period from 7 to 14 days to evaluate potential delayed effects beyond the primary exposure window.

### Software

All analyses were performed in Python version 3.8.20 using the pandas, numpy, statsmodels, patsy, and matplotlib packages. DLNMs were implemented using custom-built cross-basis functions within the statsmodels framework. Natural cubic splines were generated using the cr function from patsy. Bootstrap analyses were performed using numpy-based resampling procedures. Statistical modeling was conducted using GLM with a quasi-Poisson distribution. All statistical tests were two-sided, and a *P* value of less than 0.05 was considered statistically significant.

## Results

### Descriptive statistics of MI incidence and meteorological factors

A total of 1,461 days were included in the analysis from 2020 to 2023 in Tianjin. The mean daily number of MI events was 23.92 ± 6.05, with a maximum of 44 cases per day (Table 1). The mean daily number of STEMI cases was 14.93 ± 4.30, which was higher than that of NSTEMI cases was 8.98 ± 3.71.

**Table 1:** Descriptive statistics of MI incidence and meteorological factors

Variable	Mean ± SD	Median (IQR)	Min	Max
Total MI Cases (n)	23.92 ± 6.05	24.00 (20.00, 28.00)	6	44
STEMI Subtype (n)	14.93 ± 4.30	15.00 (12.00, 18.00)	3	30
NSTEMI Subtype (n)	8.98 ± 3.71	9.00 (6.00, 11.00)	1	24
Mean Temperature (°C)	14.2 ± 11.2	15.4 (4.2, 24.4)	-13.8	34.2
Max Temperature (°C)	20.4 ± 11.0	22.2 (10.9, 30.3)	-7.9	41.0
Min Temperature (°C)	7.9 ± 11.3	8.06 (-2.0, 18.4)	-19.9	27.5
Relative Humidity (%)	52.10 ± 23.75	53.61 (33.40, 71.67)	0.83	98.61
Wind Speed (m/s)	5.11 ± 1.99	4.70 (3.60, 6.20)	1.5	16.9

*MI* Myocardial infarction, *STEMI* ST-segment elevation myocardial infarction, *NSTEMI* Non-ST-segment elevation myocardial infarction, *IQR* Interquartile range

Meteorological conditions showed variation during the study period. The mean daily temperature was 14.2 ± 11.2 °C, ranging from − 19.9 °C to 41.0 °C. Mean relative humidity was 52.10%, and the maximum daily wind speed was 15.90 m/s.

### Correlation analysis of environmental and clinical variables

**Fig. 1:**
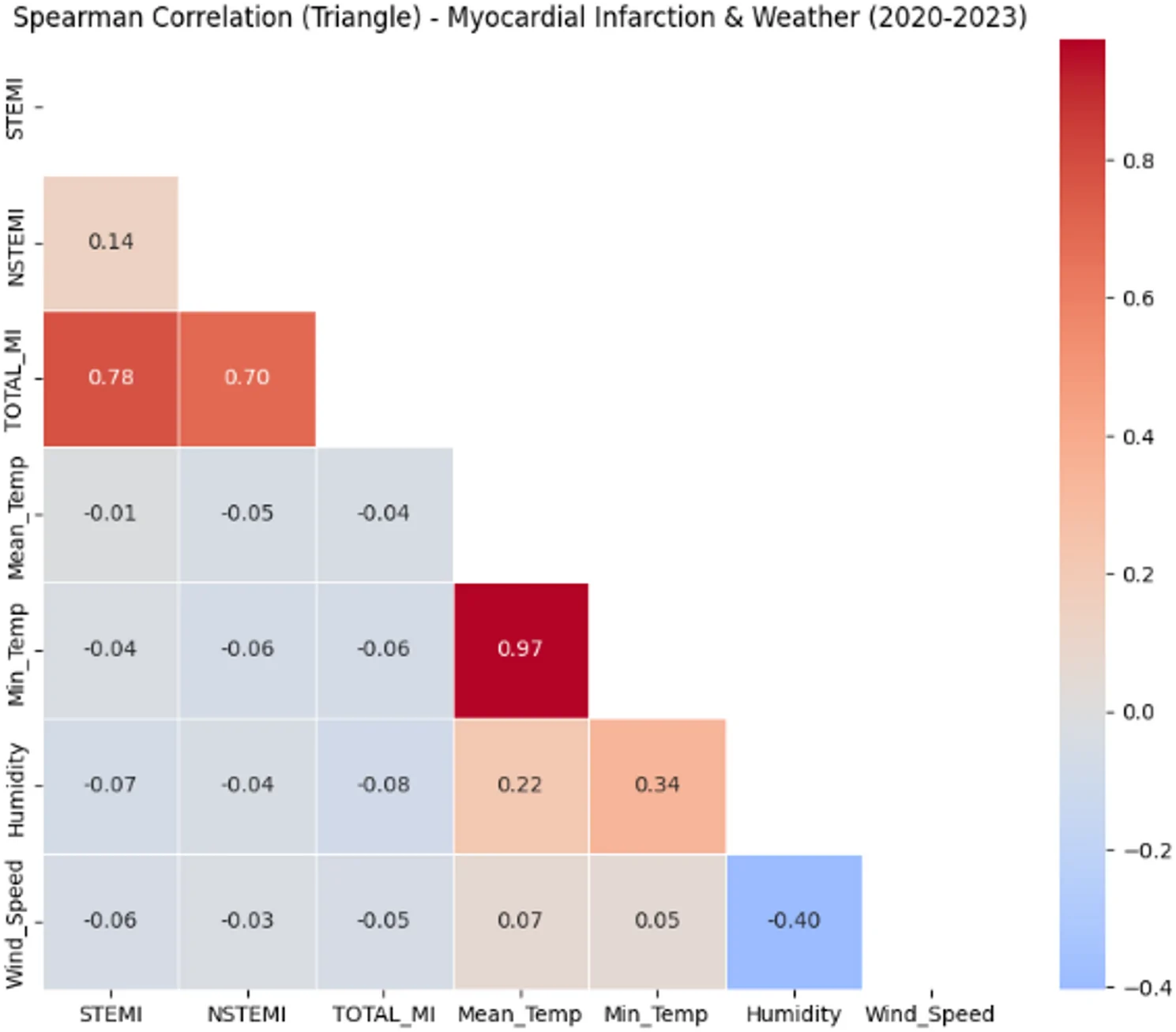
Spearman rank correlation matrix of daily MI incidence and meteorological factors. The heatmap shows pairwise Spearman correlation coefficients (r) between daily myocardial infarction (MI) cases and environmental variables in Tianjin from 2020 to 2023. Color intensity and cell values represent the magnitude and direction of correlations. Red indicates positive correlations, while blue indicates negative correlations. MI, myocardial infarction; STEMI, ST-segment elevation myocardial infarction; NSTEMI, non-ST-segment elevation myocardial infarction

Spearman rank correlation coefficients were calculated to assess associations between daily MI counts and meteorological variables (Fig. 1). Total MI was positively correlated with STEMI (*r* = 0.78) and NSTEMI (*r* = 0.70). Correlations between daily MI counts and meteorological variables were weak, with total MI showing weak negative correlations with mean temperature (*r* = − 0.04), minimum temperature (*r* = − 0.06), relative humidity (*r* = − 0.08), and wind speed (*r* = − 0.05). Similar patterns were observed for STEMI and NSTEMI.

Among meteorological variables, mean temperature and minimum temperature were highly correlated (*r* = 0.97). Relative humidity showed a moderate positive correlation with minimum temperature (*r* = 0.34) and a moderate negative correlation with wind speed (*r* = − 0.40).

### Lag-response relationship between extreme cold and the risk of MI over a 7-day period

During the study period, 34,940 MI events were recorded, including 21,814 (62.4%) STEMI cases and 13,126 (37.6%) NSTEMI cases. The main DLNM for total MI showed mild overdispersion, with a Pearson dispersion statistic of 1.12 and a deviance-to-residual degrees of freedom ratio of 1.13. A quasi-Poisson regression was applied to account for overdispersion in daily MI counts.

**Fig. 2:**
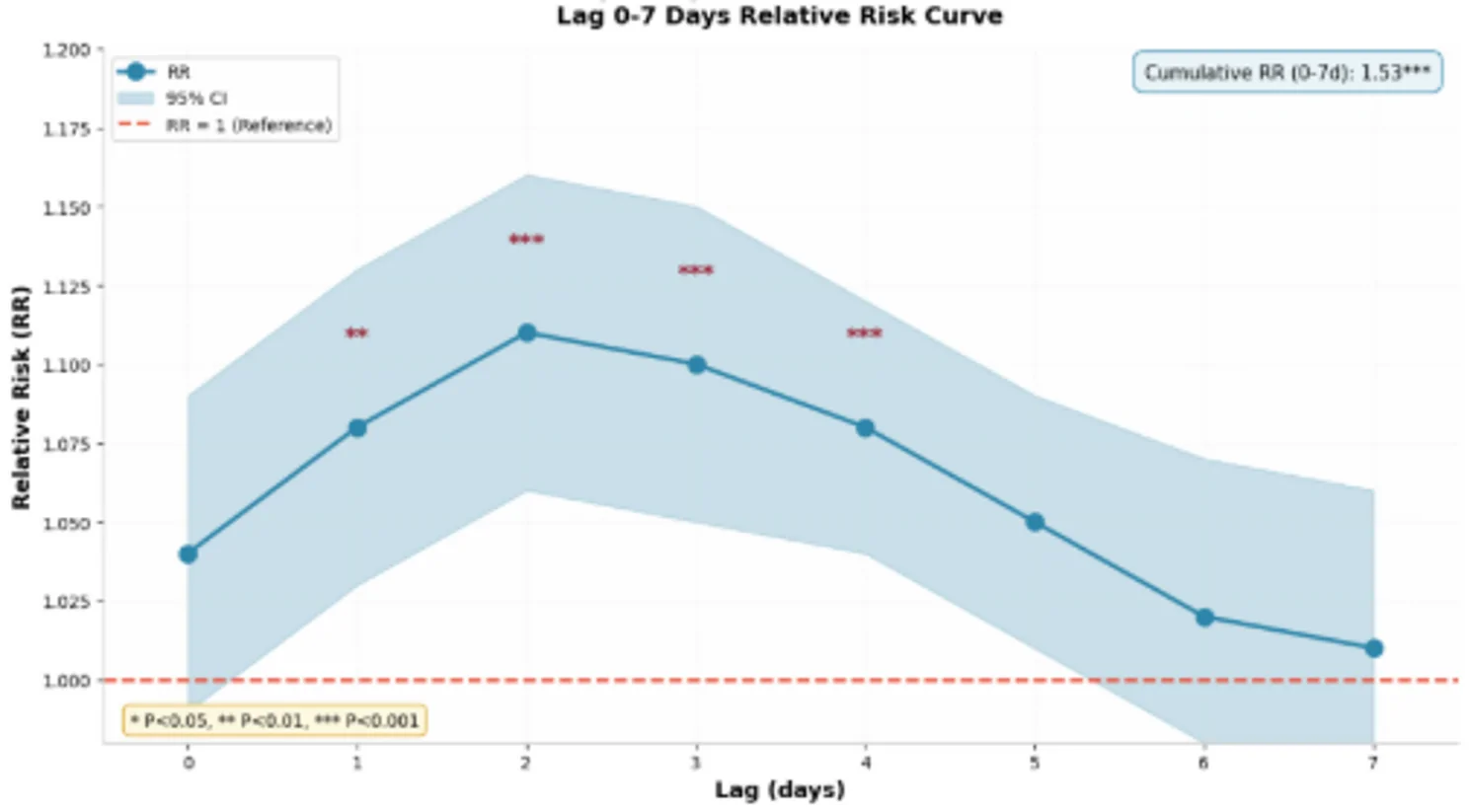
Lag-response relationship between extreme cold (−3.4 °C) and the risk of myocardial infarction over a 7-day period. The blue solid line represents the RR across different lag days (lag 0–7), with the blue shaded area indicating the 95% CI. MI, myocardial infarction; RR, relative risk；CI, confidence interval. **P<0.01, ***P<0.001

Using distributed lag non-linear models, we estimated the lag-response association between extreme cold exposure and MI risk. With the median temperature (15.4 °C) as the reference, extreme cold exposure (− 3.4 °C, 5th percentile) corresponded to a cumulative 7-day RR of 1.53 (95% CI: 1.25–1.87, *P* < 0.001) (Table 2; Fig. 2).

**Table 2:** Lag-response relationship between extreme cold and the risk of MI over a 7-day period

Lag (days)	Total MI RR [95% CI]	*P* value	STEMI RR [95% CI]	*P* value	NSTEMI RR [95% CI]	*P* value
Lag 0	1.04 [0.99, 1.09]	0.112	1.05 [0.98, 1.13]	0.162	1.02 [0.93, 1.12]	0.671
Lag 1	1.08 [1.03, 1.13]	0.002	1.09 [1.02, 1.17]	0.012	1.05 [0.96, 1.15]	0.284
Lag 2	1.11 [1.06, 1.16]	< 0.001	1.12 [1.05, 1.19]	< 0.001	1.08 [1.00, 1.17]	0.048
Lag 3	1.10 [1.05, 1.15]	< 0.001	1.10 [1.04, 1.16]	0.001	1.09 [1.02, 1.18]	0.011
Lag 4	1.08 [1.04, 1.12]	< 0.001	1.08 [1.03, 1.14]	0.003	1.07 [1.01, 1.14]	0.024
Lag 5	1.05 [1.01, 1.09]	0.058	1.05 [0.99, 1.11]	0.104	1.04 [0.98, 1.11]	0.183
Lag 6	1.02 [0.98, 1.07]	0.354	1.02 [0.97, 1.08]	0.403	1.02 [0.95, 1.09]	0.582
Lag 7	1.01 [0.97, 1.06]	0.682	1.01 [0.95, 1.07]	0.751	1.01 [0.94, 1.08]	0.814
Cumulative (0–7d)	1.53 [1.25, 1.87]	< 0.001	1.62 [1.31, 2.01]	< 0.001	1.38 [1.05, 1.82]	0.019

The median temperature (15.4 °C) was used as the reference

*Abbreviations*: *MI* Myocardial infarction, *STEMI* ST-segment elevation myocardial infarction, *NSTEMI* Non-ST-segment elevation myocardial infarction, *RR* Relative risk, *CI* Confidence interval

In the single-day lag analysis, no statistically significant association was observed at lag 0 (RR = 1.04, 95% CI: 0.99–1.09). Statistically significant associations were observed from lag 1 to lag 4: lag 1 (RR = 1.11, 95% CI: 1.06–1.16), lag 2 (RR = 1.11, 95% CI: 1.06–1.16), lag 3 (RR = 1.10, 95% CI: 1.05–1.15), and lag 4 (RR = 1.08, 95% CI: 1.04–1.12). No statistically significant associations were observed after lag 4, with estimates approaching the null at lag 7 (RR = 1.01, 95% CI: 0.97–1.06) (Table 2; Fig. 2).

### Subgroup analysis by MI subtype

Subgroup analyses were performed for STEMI and NSTEMI following exposure to extreme cold (− 3.4 °C) relative to the median temperature (15.4 °C). The cumulative 7-day RR of extreme cold exposure was 1.62 (95% CI: 1.31–2.01) for STEMI and 1.38 (95% CI: 1.05–1.82) for NSTEMI. The Wald-type heterogeneity test did not indicate a statistically significant difference between the two estimates (Z = 0.902, *P*_heterogeneity_ = 0.367).

For STEMI, statistically significant associations were observed from lag 1 to lag 4: lag 1 (RR = 1.09, 95% CI: 1.02–1.17), lag 2 (RR = 1.12, 95% CI: 1.05–1.19), lag 3 (RR = 1.10, 95% CI: 1.05–1.15), and lag 4 (RR = 1.08, 95% CI: 1.03–1.14). No significant association was observed at lag 0.

**Fig. 3:**
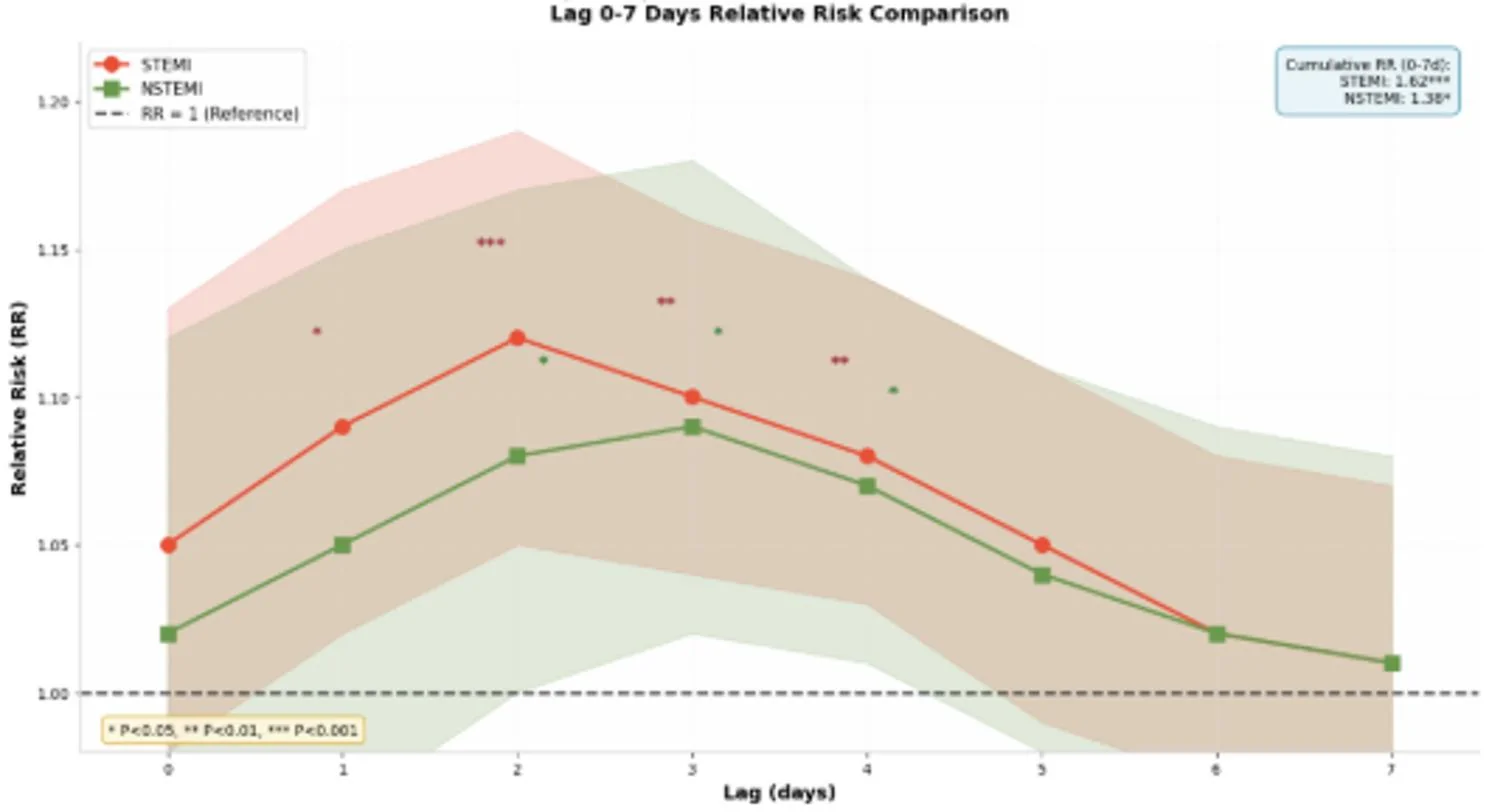
Comparison of lag-response relationships for STEMI and NSTEMI subtypes at -3.4°C. RR trajectories for STEMI (red line) and NSTEMI (green line) are shown with their corresponding 95% CI (shaded areas). STEMI, ST-segment elevation myocardial infarction; NSTEMI, non-ST-segment elevation myocardial infarction; RR, relative risk; CI, confidence interval. * *P* < 0.05, ** *P* < 0.01, *** *P* < 0.001

For NSTEMI, statistically significant associations were observed from lag 2 to lag 4: lag 2 (RR = 1.08, 95% CI: 1.00–1.17), lag 3 (RR = 1.09, 95% CI: 1.02–1.18), and lag 4 (RR = 1.07, 95% CI: 1.01–1.14). No significant associations were observed at lag 0, lag 1, or lags 5–7 (Table 2; Fig. 3).

### Sensitivity analysis using an extended 14-day lag window

To examine whether a longer lag window altered the main findings, we extended the maximum lag from 7 to 14 days in a sensitivity analysis. The overall results were consistent with the primary 7-day model.

For total MI, statistically significant associations were observed from lag 1 to lag 4, with the highest estimate at lag 2. After lag 7, the estimates were close to null (0.99–1.01) across lag 8 to lag 14, with no statistically significant associations observed in this extended period.

The cumulative association over lag 0–14 days remained significant for total MI (RR = 1.51, 95% CI: 1.20–1.90), STEMI (RR = 1.58, 95% CI: 1.23–2.03), and NSTEMI (RR = 1.35, 95% CI: 1.01–1.81). Compared with the 0–7 day model, the extended lag window did not materially change the cumulative risk estimates. For STEMI and NSTEMI, similar patterns were observed, with significant associations mainly within the first 4 days after exposure and attenuation thereafter (Supplementary Table S1).

### Non-linear cumulative exposure-response relationship between temperature and MI risk

**Fig. 4:**
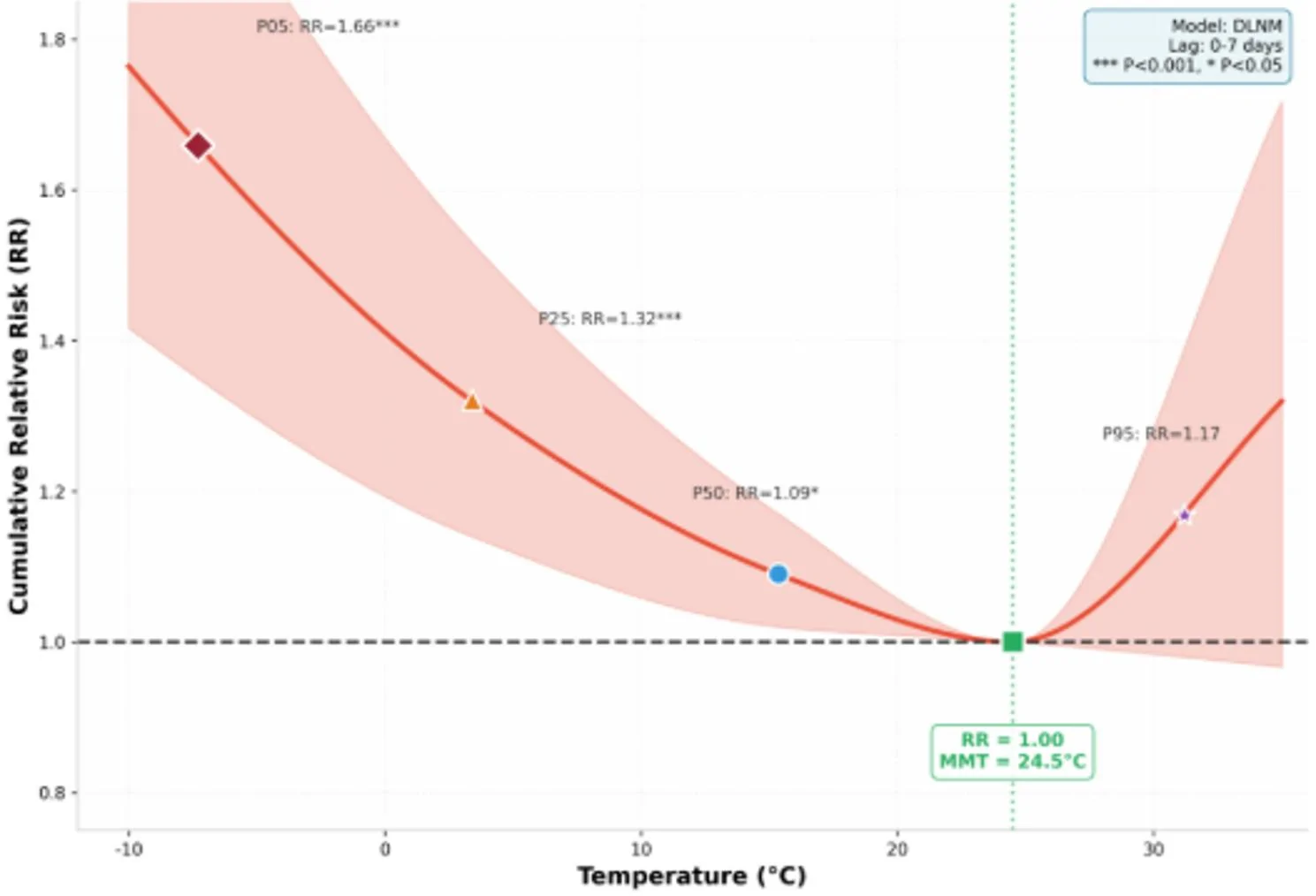
Cumulative exposure–response relationship between temperature and MI risk using the MMT as the reference. The association between temperature and MI risk was estimated using a DLNM with a lag period of 0–7 days. The solid red line represents the RR, and the shaded area indicates the 95% CI. The horizontal dashed line at RR = 1.0 indicates the reference level. The minimum morbidity temperature (MMT = 24.5 °C), corresponding to the lowest predicted MI risk, is marked by a green square (RR = 1.00). All relative risks were recalculated using the MMT as the reference. Key temperature percentiles are indicated: P05 (extreme cold, −3.4 °C; RR = 1.66, P < 0.001), P25 (3.4 °C; RR = 1.32, *P* < 0.001), P50 (15.4 °C; RR = 1.09, *P* < 0.05), and P95 (31.2 °C; RR = 1.17, *P* = 0.12). MI, myocardial infarction; MMT, minimum morbidity temperature; DLNM, distributed lag non-linear model; RR, relative risk.

To further examine the temperature–MI association, the cumulative exposure–response curve was re-estimated using the MMT as the reference category. The primary DLNM identified an MMT of 24.5 °C, corresponding approximately to the 75th percentile of the local temperature distribution. Bootstrap analysis yielded a similar estimate of 24.1 °C (95% CI: 22.6–26.2 °C), indicating that the estimated optimal temperature was robust to sampling variability (Fig. 4).

Compared with the MMT, increased MI risk was observed at lower temperatures. At the extreme cold threshold (− 3.4 °C, 5th percentile), the cumulative RR was 1.66 (95% CI: 1.35–2.04, *P* < 0.001). Elevated risks were also observed at the 25th percentile (3.4 °C; RR = 1.32, 95% CI: 1.14–1.53, *P* < 0.001) and at the median temperature (15.4 °C; RR = 1.09, 95% CI: 1.02–1.17, *P* < 0.05). In contrast, the association at the 95th percentile (31.2 °C) was not statistically significant (RR = 1.17, 95% CI: 0.98–1.39).

Sensitivity analyses using alternative reference specifications for the exposure–response curve produced consistent results.

## Discussion

This time-series analysis in Tianjin identified a short-term association between ambient cold exposure and daily MI counts. Using distributed lag non-linear models, we found that extreme cold corresponded to a cumulative 7-day RR of 1.53 relative to the median temperature, with the strongest association observed at lag 2 days. When the MMT was used as the reference, the overall temperature–response curve suggested a non-linear pattern with relatively higher risks at lower temperatures.

Moderate and extreme cold were associated with higher cumulative MI risk, whereas the heat-related estimate did not reach statistical significance, possibly due to the limited frequency and short duration of extreme heat events in this temperate monsoon setting. These findings support a short-term association between cold exposure and MI presentations in northern China, but should not be interpreted as evidence of a direct causal effect.

Our findings are broadly consistent with previous studies reporting non-linear associations between temperature and MI. In Taiwan, MI risk increased progressively as temperature fell below the local optimum [12]. In the SWEDEHEART study, MI incidence increased at lower temperatures and declined at milder temperatures [13]. A time-series study from Xuzhou also reported increased MI risk under both moderate and extreme cold conditions [14]. Together, these studies suggest that the temperature associated with the lowest cardiovascular risk may vary across regions and that local climatic conditions play an important role in shaping the exposure–response relationship. In Tianjin, where winters are prolonged and cold, the observed minimum morbidity temperature was lower than that reported in subtropical settings but still indicated a clear increase in MI risk at lower temperatures [12–14]. The location of the estimated MMT near the 75th percentile is plausible in the local climatic context. Cold exposure is frequent and prolonged during winter, and the exposure–response curve is influenced by increased MI risk at lower temperatures. In such settings, the lowest predicted risk may occur in a relatively warm part of the local temperature distribution rather than around the median. Similar patterns have been reported in previous temperature–health studies in China, where optimum temperatures for cardiovascular or mortality outcomes often occur at upper percentiles of the local temperature distribution. Therefore, the MMT in this study should be viewed as a region-specific empirical reference point rather than a universal physiological threshold.

The lag pattern observed in our study is also consistent with earlier reports. We found that the effect of extreme cold did not appear on the day of exposure, became evident on the following day, peaked at lag 2, and gradually declined thereafter. A nationwide study in 323 Chinese cities reported that cold-spell effects on acute MI peaked around day 3 and dissipated within several days [15]. In Sweden, short-term temperature decreases were likewise associated with delayed increases in MI admissions [3]. The Xuzhou study also suggested that cold exposure could trigger excess MI risk over subsequent days [14]. Taken together, these findings suggest that cold-related short-term associations with MI are not purely immediate, and that relatively short lag structures may be relevant when considering cold-related health risk management and preparedness strategies.

A descriptive divergence in the lag-response patterns between STEMI and NSTEMI was observed in our subgroup analysis. The point estimate of the cumulative 7-day relative risk appeared higher for STEMI, with a slightly earlier and more pronounced temporal pattern compared with NSTEMI. Similar observations have been reported in previous cohort studies, including those conducted in Nordic populations [3, 13]. However, it is important to emphasize that the formal heterogeneity test did not demonstrate a statistically significant difference between the two subtypes (*P*_heterogeneity_ = 0.367). Therefore, these subgroup differences should be interpreted as descriptive patterns rather than evidence of distinct susceptibility.

From a mechanistic perspective, previous experimental and epidemiological studies have suggested that acute cold exposure may activate sympathetic nervous system responses, leading to peripheral vasoconstriction, transient increases in blood pressure, and changes in hemorheological properties [16–22]. These physiological responses have been proposed to contribute to acute cardiovascular stress and may manifest differently across acute coronary syndrome subtypes. However, given the ecological design of the present study and the absence of individual-level clinical data, causal or mechanistic interpretations cannot be confirmed. The observed differences between STEMI and NSTEMI therefore remain hypothesis-generating and require further investigation in studies with more detailed clinical and biological measurements.

The magnitude of the estimated cold-related vulnerability in Tianjin warrants detailed consideration. Relative to the MMT, moderate and extreme cold temperatures were associated with a 32% and 66% excess risk of MI, respectively. These observations are broadly consistent with findings from both domestic and international cohorts, although effect sizes vary across regions [12, 14, 15, 23, 24]. For example, in Houston, ambient cold was found to have a more pronounced relationship with MI than heat [23], whereas a population-based study in Lithuania documented more modest risk elevations during winter cold spells [24]. Such heterogeneity across geographic regions is likely related to differences in baseline climate, building insulation standards, population age structures, and behavioral adaptation strategies (e.g., central heating infrastructure). This context-specific nature highlights the importance of considering local environmental conditions when interpreting temperature–health associations, rather than directly transferring estimates across regions.Our findings support ambient cold exposure as a relevant environmental marker for short-term increases in MI presentations in northern China. The short lag structure and the relatively higher risk observed for STEMI suggest potential value for cold-weather early warning systems and targeted risk communication. During periods of rapid temperature decline, enhanced awareness and preparedness strategies may help support cardiovascular risk management in vulnerable populations, particularly those with established coronary artery disease.

This study has several limitations that should be considered when interpreting the findings. First, the ecological time-series design based on aggregated daily data reflects population-level associations and does not allow causal inference at the individual level, introducing the possibility of ecological fallacy. Second, individual-level demographic and clinical characteristics, including age, sex, comorbidities, smoking status, medication use, and socioeconomic factors, were not available, limiting the ability to identify vulnerable subpopulations or assess effect modification. Third, the lack of adjustment for ambient air pollutants (PM₂.₅, NO₂, O₃), which are established time-varying cardiovascular risk factors, may introduce residual confounding. However, the direction and magnitude of this bias remain uncertain due to context-specific correlations with temperature. Fourth, the registry captures hospital-managed MI cases within the CPC network and does not include out-of-hospital deaths or non-CPC hospital presentations, which may lead to selection bias and underestimation of the total population burden. Fifth, exposure assessment relied on a single central meteorological monitoring station, which may not fully capture spatial heterogeneity in ambient temperature across the study region, leading to potential non-differential exposure misclassification in this time-series design.

Sixth, the study period overlaps with the COVID-19 pandemic, during which healthcare utilization patterns may have changed, and no formal stratified sensitivity analysis was conducted. Seventh, the MMT is a model-derived estimate and should be interpreted as a context-specific reference rather than a fixed biological threshold. Finally, heat-related associations may be limited by reduced statistical power and should be interpreted cautiously. Future studies integrating individual-level clinical data, high-resolution environmental exposures, and stratified analyses across demographic and temporal subgroups are warranted to further clarify susceptible populations and disentangle the independent effects of temperature and co-exposures on myocardial infarction risk.

In conclusion, ambient cold exposure was associated with a transient increase in myocardial infarction risk in Tianjin, with the strongest effect observed approximately 2 days after exposure. Although STEMI showed higher point estimates than NSTEMI, no statistically significant heterogeneity was found. Cold-related associations were evident, whereas heat-related effects were not statistically significant, possibly due to limited statistical power; thus, the absence of a heat effect should not be interpreted as no risk. As the study is based on hospital-managed cases, results may not fully reflect population-level incidence or allow identification of vulnerable subgroups. These findings may support cold-weather cardiovascular risk management in similar temperate regions.

## Supplementary Information

Supplementary Material 1.

Supplementary Material 2.

## Data Availability

The clinical data used in this study are not publicly available because they contain potentially identifiable patient information, but are available from the corresponding author on reasonable request and with permission from the relevant institution.

## Abbreviations

MI: Myocardial infarction
MMT: Minimum morbidity temperature
RR: Relative risk
CI: Confdential intervals
STEMI: ST-segment elevation myocardial infarction
NSTEMI: Non-ST-segment elevation myocardial infarction
CVD: Cardiovascular disease
RH: Relative humidity
SD: Standard Deviation
IQR: Interquartile Range
DLNM: Distributed Lag Non-linear Model
GLM: Generalized linear model
CPC: Chest Pain Center
