# Supplementary material for: Ambient cold exposure and myocardial infarction risk in Tianjin, China: a distributed lag non-linear analysis
Source: BMC Public Health. 2026 Aug 7;26:2675. doi: 10.1186/s12889-026-28924-7 (PMC13602570; doi:10.1186/s12889-026-28924-7)
Supplement: Supplementary file 1 [file 12889_2026_28924_MOESM1_ESM.docx]

| **Table S1 Lag-response relationship between extreme cold and the risk of MI over a 14-day period** | | | | | | |
| --- | --- | --- | --- | --- | --- | --- |
| Lag (days) | Total MI  RR [95% CI] | P value | STEMI  RR [95% CI] | P value | NSTEMI  RR [95% CI] | P value |
| Lag 0 | 1.04 [0.99, 1.09] | 0.112 | 1.05 [0.98, 1.13] | 0.162 | 1.02 [0.93, 1.12] | 0.671 |
| Lag 1 | 1.08 [1.03, 1.13] | 0.002 | 1.09 [1.02, 1.17] | 0.012 | 1.05 [0.96, 1.15] | 0.284 |
| Lag 2 | 1.11 [1.06, 1.16] | <0.001 | 1.12 [1.05, 1.19] | <0.001 | 1.08 [1.00, 1.17] | 0.048 |
| Lag 3 | 1.10 [1.05, 1.15] | <0.001 | 1.10 [1.04, 1.16] | 0.001 | 1.09 [1.02, 1.18] | 0.011 |
| Lag 4 | 1.08 [1.04, 1.12] | <0.001 | 1.08 [1.03, 1.14] | 0.003 | 1.07 [1.01, 1.14] | 0.024 |
| Lag 5 | 1.05 [1.01, 1.09] | 0.058 | 1.05 [0.99, 1.11] | 0.104 | 1.04 [0.98, 1.11] | 0.183 |
| Lag 6 | 1.02 [0.98, 1.07] | 0.354 | 1.02 [0.97, 1.08] | 0.403 | 1.02 [0.95, 1.09] | 0.582 |
| Lag 7 | 1.01 [0.97, 1.06] | 0.682 | 1.01 [0.95, 1.07] | 0.751 | 1.01 [0.94, 1.08] | 0.814 |
| Lag 8 | 1.00 [0.95, 1.05] | 0.912 | 1.00 [0.93, 1.07] | 0.954 | 1.00 [0.92, 1.08] | 0.931 |
| Lag 9 | 0.99 [0.94, 1.04] | 0.741 | 0.99 [0.92, 1.06] | 0.812 | 1.00 [0.91, 1.09] | 0.962 |
| Lag 10 | 0.99 [0.93, 1.05] | 0.682 | 0.98 [0.91, 1.06] | 0.611 | 0.99 [0.90, 1.10] | 0.894 |
| Lag 11 | 1.00 [0.94, 1.06] | 0.943 | 0.99 [0.92, 1.07] | 0.824 | 1.00 [0.91, 1.11] | 0.951 |
| Lag 12 | 1.00 [0.95, 1.06] | 0.971 | 1.00 [0.93, 1.08] | 0.912 | 1.01 [0.92, 1.11] | 0.812 |
| Lag 13 | 1.01 [0.95, 1.07] | 0.762 | 1.01 [0.94, 1.09] | 0.714 | 1.01 [0.92, 1.12] | 0.793 |
| Lag 14 | 1.00 [0.94, 1.06] | 0.985 | 1.00 [0.93, 1.08] | 0.991 | 1.00 [0.91, 1.11] | 0.974 |
| Cumulative (0–14d) | 1.51 [1.20, 1.90] | <0.001 | 1.58 [1.23, 2.03] | <0.001 | 1.35 [1.01, 1.81] | 0.042 |

Abbreviations: MI, myocardial infarction; STEMI, ST-segment elevation myocardial infarction; NSTEMI, non-ST-segment elevation myocardial infarction; RR, relative risk; CI, confidence interval.
