# Supplementary material for: Ambient cold exposure and myocardial infarction risk in Tianjin, China: a distributed lag non-linear analysis
Source: BMC Public Health. 2026 Aug 7;26:2675. doi: 10.1186/s12889-026-28924-7 (PMC13602570; doi:10.1186/s12889-026-28924-7)
Supplement: Supplementary file 2 [file 12889_2026_28924_MOESM2_ESM.docx]

**Supplementary Figure S1** Distribution of daily mean temperatures in Tianjin (2020–2023).

**Figure Legends**

**Supplementary Figure S1** Distribution of daily mean temperatures in Tianjin (2020–2023).Vertical dashed lines indicate the 5th, 25th, 50th (median), 75th (corresponding approximately to the minimum morbidity temperature, MMT), and 95th percentiles of daily mean temperatures. The MMT of 24.5°C represents the temperature associated with the lowest cumulative risk of myocardial infarction in this population and corresponds approximately to the 75th percentile of the local temperature distribution.
